# Supplementary figures and images for: Genome sequencing and multi-stage, blood-feeding, and tissue-specific transcriptome atlas of the Rocky Mountain wood tick provide a critical resource for this vector
Source: bioRxiv. 2026 Apr 17:2026.04.15.717773. Preprint. [Version 1] doi: 10.64898/2026.04.15.717773 (PMC13105059; doi:10.64898/2026.04.15.717773)

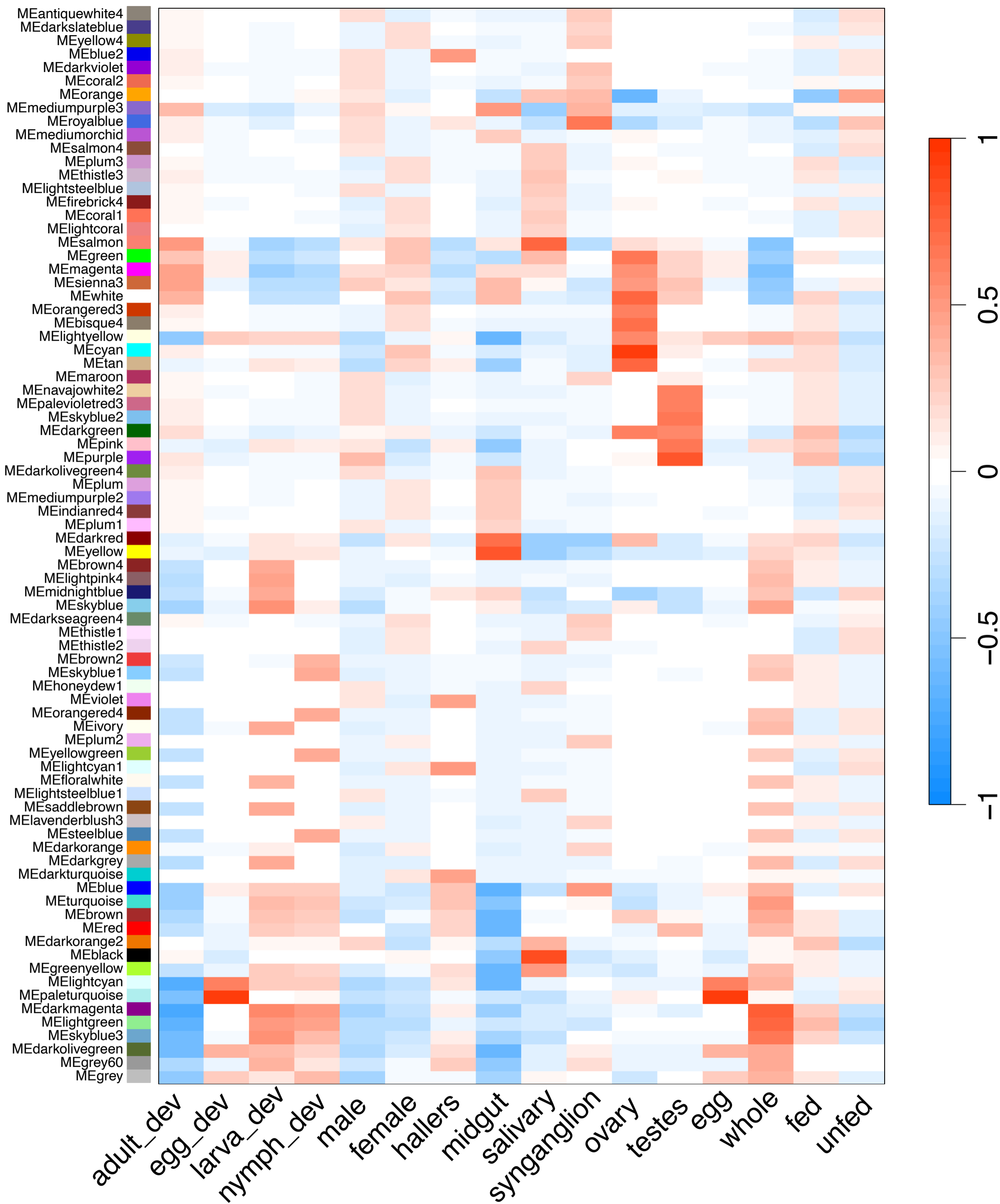

Supplement: Supplement 1 — Figure S1 - Weighted Gene Co-expression Network Analysis (WGCNA) was used to identify modules of co-expressed genes across multiple tissues and developmental stages of Dermacentor andersoni. [file media-1.pdf]
